# Active Exsanguination Behind a Urinary Tract Infection (UTI) Diagnostic Label: A Quadruple-Hit Cardiorenal Collapse Following Ambulatory Urological Care

**DOI:** 10.7759/cureus.118182

**Published:** 2026-10-19

**Authors:** Wutt Oo

**Affiliations:** 1 Emergency Department, Broomfield Hospital, Broomfield, GBR

**Keywords:** collapse, haematuria, severe anaemia, st-segment elevation myocardial infarction (stemi), trifascicular block

## Abstract

We report the case of a 95-year-old man with a previous history of myocardial infarction, pulmonary oedema, and hypertension who developed recurrent collapse while undergoing ambulatory urological follow-up for a six-week history of macroscopic haematuria. Following a recent outpatient review, he had received oral antibiotics for a presumed urinary tract infection (UTI). Throughout this six-week ambulatory period, no full blood count or other blood tests were performed, leaving the extent of blood loss and its haematological consequences clinically occult.

The patient subsequently experienced three episodes of sudden collapse within one week at home. The episodes were preceded by a prodrome of marked weakness. On assessment by ambulance clinicians, a profound postural blood-pressure drop was identified. Given the postural component and presumed recent urinary infection, dehydration was considered the initial explanation, and intravenous fluid resuscitation was commenced. However, hospital point-of-care investigations substantially changed the diagnostic picture. Venous blood gas analysis demonstrated a haemoglobin concentration consistent with profound anaemia. ECG demonstrated a right bundle branch block, left axis deviation, and a prolonged PR interval, indicating significant conduction-system disease in the context of previous ischaemic myocardial injury. Serum biochemistry demonstrated elevated creatinine and urea, alongside a severely reduced estimated glomerular filtration rate, consistent with severe renal dysfunction. Venous blood gas analysis further showed a low pH and elevated lactate, indicating significant metabolic acidosis.

The clinical picture suggested an interaction between severe blood-loss anaemia, impaired cardiovascular compensatory reserve, postural haemodynamic stress, and renal hypoperfusion. Severe anaemia substantially reduces arterial oxygen content, necessitating compensatory increases in cardiac output and oxygen extraction. When cardiovascular reserve is already compromised, this compensation may be inadequate. Emergency packed red blood cell transfusion and careful fluid-volume optimisation were undertaken. Given the recurrent collapse and significant conduction abnormalities, an urgent cardiology transfer was arranged for permanent pacemaker insertion. Severe renal dysfunction prompted urgent nephrology involvement and transfer.

## Introduction

Syncope is caused by transient global cerebral hypoperfusion and represents a common clinical presentation with a broad differential diagnosis [1,2]. Orthostatic and neurally mediated mechanisms are frequent, but cardiac causes, haemorrhage, and other systemic conditions must be considered according to the clinical context. The initial assessment should include a detailed history, physical examination, including lying and standing blood-pressure measurements, and a standard electrocardiogram. Blood tests should be performed when clinically indicated, including haemoglobin or haematocrit when haemorrhage is suspected.

The diagnostic challenge becomes greater in older adults because physiological reserve is reduced and multimorbidity is common. Orthostatic hypotension is particularly prevalent in older patients and may be related to impaired autonomic responses, cardiovascular disease, medications, reduced circulating volume, or a combination of these factors [3]. Consequently, the identification of a postural blood-pressure reduction should be regarded as a physiological finding rather than a definitive diagnosis of an autonomic disorder.

Anaemia adds another important dimension to circulatory physiology. Oxygen delivery to tissues depends on cardiac output and arterial oxygen content, the latter being strongly influenced by haemoglobin concentration. Severe reductions in haemoglobin therefore require cardiovascular compensation to maintain tissue oxygen delivery. In a patient with previous myocardial infarction or impaired cardiac reserve, the capacity to increase cardiac output may be restricted, making profound anaemia substantially more dangerous [4]. The relationship between anaemia and myocardial disease is particularly important. Acute anaemia reduces oxygen delivery to the myocardium and can worsen the imbalance between myocardial oxygen supply and demand, particularly in patients with established coronary disease. Thus, profound anaemia in a patient with previous ST-segment elevation myocardial infarction (STEMI) should not be regarded simply as a laboratory abnormality; it may become a major haemodynamic and cardiovascular stressor.

Cardiac conduction disease can further reduce compensatory capacity. The 2018 American College of Cardiology, American Heart Association, and Heart Rhythm Society (ACC/AHA/HRS) guideline emphasizes the importance of ECG abnormalities in patients with suspected conduction disease and notes that bundle branch block in patients with syncope may indicate significant underlying conduction-system disease. In patients with structural heart disease or previous myocardial infarction, the possibility of intermittent high-grade atrioventricular block must be considered when unexplained collapse occurs [5,6]. The renal system is also vulnerable to systemic haemodynamic compromise. Acute kidney injury (AKI) may develop when renal perfusion is inadequate, particularly in the setting of hypotension, reduced effective circulating volume, or other systemic insults. Severe AKI may then further worsen the patient’s physiological state through accumulation of metabolic waste and disturbances in acid-base and electrolyte balance.

The patient’s persistent macroscopic haematuria represented another important diagnostic issue. Visible haematuria in older adults requires careful evaluation because it can be associated with significant underlying urological pathology, including urinary tract malignancy [7]. National Institute for Health and Care Excellence (NICE) guidelines recommend suspected cancer pathway referral for adults aged 45 years or older with visible haematuria that is unexplained or persists or recurs after successful treatment of urinary tract infection (UTI) [8].

However, this case raises a further issue beyond the need to investigate the cause of haematuria: the physiological consequences of ongoing bleeding also need to be recognised [9]. During the patient’s six-week ambulatory follow-up, no full blood count (FBC) or other blood tests were performed. Consequently, the severity of the anaemia remained undetected until the patient presented following recurrent collapse with a haemoglobin concentration of only 50 g/L. This represents a critical missed opportunity in the clinical trajectory. It would be inappropriate to claim that an FBC is mandatory at every haematuria appointment; however, in a 95-year-old patient with six weeks of ongoing macroscopic haematuria, the absence of any blood-test assessment meant that clinically significant blood loss could not be identified before the patient deteriorated.

The present case report describes how an initially plausible UTI diagnosis, combined with a lack of haematological assessment during prolonged ambulatory follow-up, allowed severe anaemia to remain unrecognised. When collapse eventually occurred, the patient was found to have not one isolated abnormality but a combination of profound anaemia, postural hypotension, significant conduction disease, severe AKI and metabolic acidosis.

## Case presentation

A 95-year-old man with a past medical history of STEMI, previous pulmonary oedema, and hypertension was undergoing active urological follow-up for a six-week history of macroscopic haematuria. There was no known history of diabetes mellitus, and his current medications are listed in Table 1.

**Table 1:** Current medications

Medications	Doses	Frequency
Loratadine	10 mg	Once daily
Sacubitril/valsartan	24/26 mg	Twice daily
Aspirin	75 mg	Once daily
Atorvastatin	40 mg	Once daily
Bumetanide	2 mg	Once daily
GTN spray	1-2 sprays	As needed
Lansoprazole	15 mg	Once daily
Spironolactone	25 mg	Once daily

The haematuria persisted during this period. The patient was followed in the ambulatory setting and had been prescribed oral antibiotics for presumed UTI following cystoscopy. The initial diagnostic framework therefore centred on urinary infection as the explanation for the continuing urinary symptoms. The major problem in the subsequent course was that no FBC or other blood tests were performed during ambulatory follow-up. Haemoglobin was therefore not measured to assess whether the persistent visible haematuria had resulted in clinically important blood loss. Renal function and metabolic consequences were also not assessed by blood tests during that period.

This absence of laboratory assessment is particularly important in retrospect. The patient ultimately presented with a haemoglobin concentration of 50 g/L and severe renal dysfunction. Although the precise rate and duration of blood loss could not be established, the combination of persistent macroscopic haematuria and profound anaemia raised serious concern about clinically significant ongoing or cumulative blood loss. The diagnostic significance of visible haematuria in an older adult is well established. The American College of Physicians recommends urological evaluation for adults with gross haematuria, even when it is self-limited, because gross haematuria can be associated with clinically important urinary tract pathology. NICE [10] similarly recommends an urgent suspected cancer pathway referral for patients aged 45 years or older with unexplained visible haematuria, or with visible haematuria that persists after successful UTI treatment.

Within one week, the patient experienced three episodes of sudden collapse at home. These episodes were associated with a prodrome of profound weakness. When the ambulance crew assessed him, they identified a marked postural drop in blood pressure. The postural hypotension led to the initial assumption that dehydration was responsible for the collapse. Intravenous fluid resuscitation was therefore commenced. Although volume depletion can cause postural hypotension, particularly in older adults, it may have multiple causes, including blood loss, medication effects, autonomic dysfunction, and cardiovascular disease.

The patient’s history, however, contained several features that should have prompted a broader assessment. He was 95 years old, had previous STEMI and pulmonary oedema, and had experienced persistent macroscopic haematuria for six weeks without documented blood-test monitoring. These features meant that significant blood loss, impaired cardiac reserve, and cardiac conduction disease all required consideration. On arrival at hospital, immediate point-of-care investigations revealed a critical abnormality. A venous blood gas demonstrated a haemoglobin concentration of 50 g/L (Table 2). This profound anaemia provided a major physiological explanation for his weakness and recurrent collapse [11].

**Table 2:** Immediate venous blood gas test showing low Hb of 50 g/L Hb: haemoglobin

Parameter	Measured value	Unit	Reference range	Status
Measured (37.0 °C)				
pH	7.25		7.31–7.41	Low
pCO_2_	5.0	kPa	—	Normal
pO_2_	3.2	kPa	—	Normal
Na+ (sodium)	133	mmol/L	130–148	Normal
K+ (potassium)	5.8	mmol/L	3.5–5.9	Normal
Cl- (chloride)	103	mmol/L	98–106	Normal
Ca++ (ionized calcium)	1.25	mmol/L	1.13–1.32	Normal
Glu (glucose)	7.8	mmol/L	3.7–8.0	Normal
Lac (lactate)	4.9	mmol/L	0.5–2.0	High
Co-oximetry				
tHb (total haemoglobin)	50	g/L	120–180	Low
Oxyhaemoglobin	19.7	%	—	Normal
CO-Hb (carboxyhaemoglobin)	0.4	%	1.5 (Max)	Normal
MetHb (methaemoglobin)	0.2	%	1.5 (Max)	Normal
HHb (deoxyhaemoglobin)	79.7	%	—	Normal
sO_2_ (oxygen saturation)	19.8	%	—	Normal
Derived parameters				
BEecf (base excess)	-11.0	mmol/L	—	Out of range
BE(B) (base excess blood)	-10.1	mmol/L	—	Out of range
Ca++(7.4)	1.18	mmol/L	—	Normal
AG (anion gap)	20	mmol/L	—	Out of range
HCO3-(c) (bicarbonate)	16.2	mmol/L	—	Out of range
HCO3- std	16.3	mmol/L	23.0–29.0	Low
Hct(c) (haematocrit)	16	%	—	Out of range

The haemoglobin concentration was particularly significant given the ongoing macroscopic haematuria. The absence of an FBC during the preceding six weeks meant that there had been no earlier objective measurement of the patient's haemoglobin. Consequently, the progression from persistent visible haematuria to severe anaemia was not recognised until the patient developed recurrent collapse.

Severe anaemia markedly reduces arterial oxygen content. The normal physiological response involves increased cardiac output and greater extraction of oxygen by tissues. However, this compensation becomes increasingly difficult when myocardial reserve is reduced. A patient with previous STEMI may have impaired ventricular function or residual myocardial damage, which can limit the ability to increase cardiac output in response to severe anaemia. The ECG provided an additional major diagnostic clue. It demonstrated right bundle branch block, left axis deviation, and a prolonged PR interval (Figures 1-2). This combination is often described clinically as "trifascicular block", which indicates significant conduction-system abnormalities in a patient presenting with recurrent collapse.

**Figure 1:**
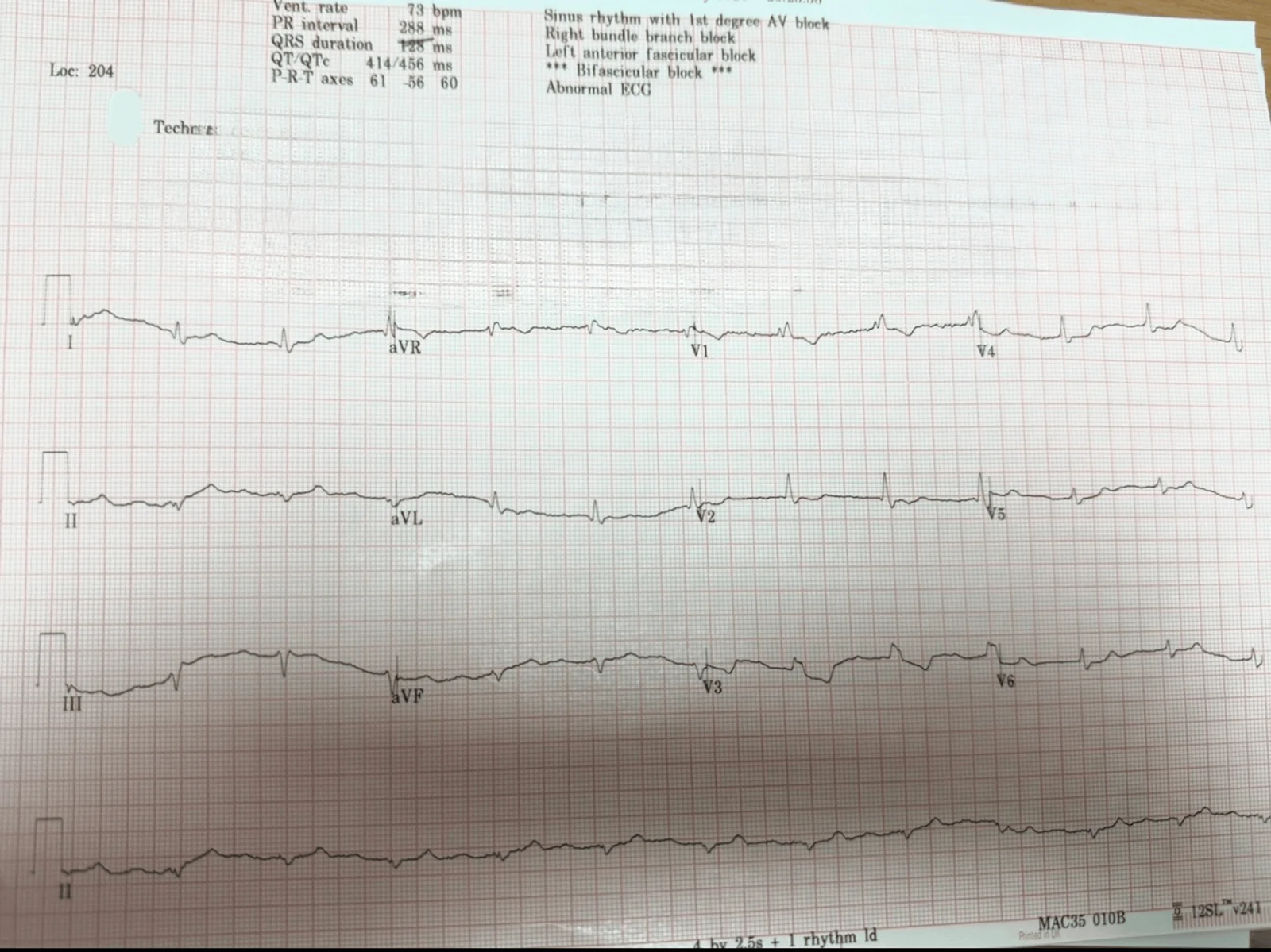
Immediate ECG showing trifascicular block ECG: electrocardiogram

**Figure 2:**
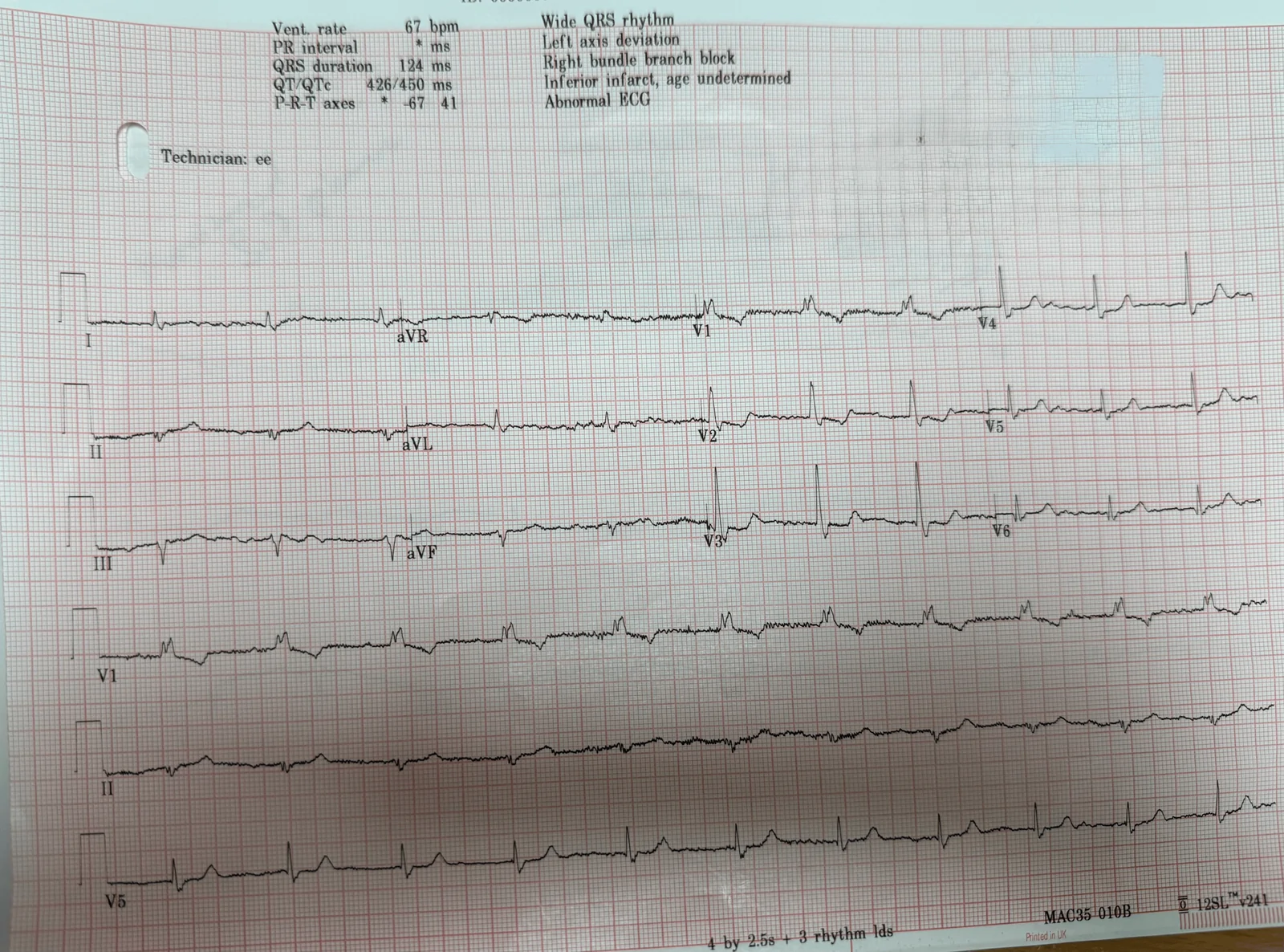
Repeated ECG within 30 minutes showing similar trifascicular block ECG: electrocardiogram

The 2018 ACC/AHA/HRS guideline identifies bundle branch block and other conduction abnormalities as clinically relevant findings in patients presenting with syncope. In patients with syncope and bundle branch block, significant His-Purkinje disease may be present, and permanent pacing may be appropriate in selected patients with documented significant conduction disease. The patient's previous STEMI increased the clinical importance of these findings. Myocardial infarction can result in both structural myocardial dysfunction and conduction-system abnormalities. Therefore, the patient's heart was potentially being asked to increase its output substantially to compensate for severe anaemia while also functioning with limited electrical and mechanical reserve.

The biochemical profile revealed another major component of the presentation (Table 3). These findings indicated severe renal dysfunction consistent with stage 3 AKI in the appropriate acute clinical context.

**Table 3:** Renal function parameters and AKI (eGFR, urea, creatinine, and electrolytes) eGFR: estimated glomerular filtration rate; acute kidney injury

Parameters	Values	Reference range	Unit
eGFR result	12	> 60	mL/min/1.73m²
Sodium	136	133–146	mmol/L
Potassium	5.7	3.5–5.3	mmol/L
Urea	39.4	2.5–7.8	mmol/L
Creatinine	363	59–104	mmol/L
AKI warning stage: 3			

AKI can develop as a consequence of renal hypoperfusion. The combination of postural hypotension reduced effective circulating volume, severe anaemia, and impaired cardiac output provides a plausible physiological pathway for reduced renal blood flow. The kidneys may therefore have become another end organ affected by the patient’s systemic circulatory compromise [12,13]. Venous blood gas showed pH 7.25, bicarbonate 16.3 mmol/L, and lactate 4.9 mmol/L, demonstrating significant metabolic acidosis with elevated lactate. Lactate may rise through several mechanisms and should not be interpreted as a specific marker of tissue hypoxia or sepsis in isolation. In this patient, however, the elevated lactate occurred alongside profound anaemia, postural hypotension, conduction disease and severe AKI, making systemic physiological stress an important consideration.

The results therefore substantially changed the original diagnostic interpretation. The presentation could no longer reasonably be explained by simple dehydration or uncomplicated UTI alone. The physiological interaction can be conceptualised as follows: Persistent macroscopic haematuria >> unrecognised blood loss >> severe anaemia >> reduced oxygen-carrying capacity >> increased cardiac demand >> limited by myocardial/ conduction disease >> postural hypotension and collapse >> renal hypoperfusion >> severe AKI and metabolic disturbance. This represented the central quadruple-hit mechanism of the case.

Major diagnostic delay: failure to assess the blood-related consequences of haematuria

The most important patient-safety issue in this case was not simply that UTI was considered. The more significant issue was that the patient had gone six weeks with ongoing macroscopic haematuria without an FBC or other blood-test assessment. The absence of an FBC meant that clinicians had no objective measurement of whether the ongoing haematuria was producing significant anaemia. When the patient ultimately collapsed, his haemoglobin was found to be 50 g/L.

**Table 4:** Laboratory FBC test showing low Hb of 50 g/L FBC: full blood count; HB: haemoglobin; CRP: C-reactive protein

Tests	Results	Reference range	Unit
CRP	1	< 5	mg/L
Haemoglobin	50	130–170	G/L
White cell count	12.6	4–10	10^9^/L
Platelets	307	150–450	10^9^/L

This distinction is important. Persistent haematuria and profound anaemia are not necessarily synonymous, and not every patient with haematuria will develop severe blood loss. Nevertheless, in a very elderly patient with persistent visible haematuria, the absence of any haematological assessment creates a safety gap, because significant anaemia can remain clinically silent until physiological compensation fails.

The European Society of Cardiology guidance on syncope recommends blood testing when clinically indicated and gives haemoglobin or haematocrit as examples when haemorrhage is suspected. NICE [14] guidance for transient loss of consciousness similarly recommends checking haemoglobin when anaemia or bleeding is suspected. The important lesson is therefore not that every routine haematuria review requires an FBC, but that persistent visible haematuria should trigger continued clinical assessment. If the bleeding continues for weeks, especially in a 95-year-old patient, clinicians should consider whether blood loss has become clinically consequential.

Furthermore, the absence of other blood tests meant that renal dysfunction also remained undetected until hospital presentation. NICE's [15] current quality standard for visible haematuria states that assessment should include renal-function testing with urea and electrolytes if this has not already been performed at referral. The delayed recognition was therefore potentially multidimensional: the patient was found to have not only severe anaemia late, but also severe renal dysfunction and metabolic disturbance that had not been identified during the preceding ambulatory period. This is a central feature of the case and should be regarded as a missed diagnostic opportunity rather than merely an abnormal laboratory finding discovered after admission.

The quadruple-hit physiological model

The first component was the haematological hit. A haemoglobin concentration of 50 g/L represents profound anaemia. Haemoglobin is a major determinant of arterial oxygen content, and severe anaemia can therefore substantially reduce oxygen delivery to tissues. The physiological responses to reduced oxygen-carrying capacity involve increased cardiac output and increased oxygen extraction. The patient, however, had a previous STEMI and pulmonary oedema. These conditions suggested pre-existing cardiovascular vulnerability. In a patient with limited myocardial reserve, the increase in cardiac output required to compensate for profound anaemia may not be achievable.

The second component was the haemodynamic hit. The patient had a profound postural drop in blood pressure. When a person stands, venous pooling reduces venous return and stroke volume. The autonomic nervous system normally compensates through vasoconstriction and an increase in heart rate. Age-related changes and cardiovascular disease can impair these compensatory mechanisms. In this patient, postural stress occurred against a background of severe anaemia and significant cardiac disease. Even if dehydration had been present, it was unlikely to have been the complete explanation. The postural blood-pressure drop may instead be viewed as the final haemodynamic stressor imposed on a circulation that had already lost substantial reserve.

The third component was the electrical and myocardial hit. The ECG showed RBBB, left axis deviation, and a prolonged PR interval. The important clinical point was not simply the label applied to the ECG but the presence of significant conduction-system disease in a patient with recurrent collapse. NICE identifies conduction abnormalities, including bundle branch block or heart block, as red flags following transient loss of consciousness that require urgent cardiovascular assessment. The ACC/AHA/HRS guideline similarly emphasises the significance of conduction abnormalities in patients with syncope [14].

A previous STEMI further increased concern because myocardial infarction can produce permanent structural and electrical abnormalities. The patient's heart therefore had potentially limited ability to increase rate and cardiac output at precisely the time when severe anaemia required greater circulatory compensation.

The fourth component was the renal and metabolic hit. A creatinine of 363 micromol/L, urea of 39.4 mmol/L, and eGFR of 12 mL/min indicated severe renal dysfunction. AKI is frequently multifactorial and may result from reduced renal perfusion, intrinsic renal injury, or obstruction.

In this case, haemodynamic compromise provided a plausible contributor. Severe anaemia reduced oxygen delivery, while postural hypotension and impaired cardiac compensation reduced effective renal perfusion. Renal dysfunction then created additional metabolic stress. The severe uraemia and metabolic acidosis were therefore not isolated laboratory abnormalities. They represented evidence that the systemic physiological disturbance had progressed to significant end-organ dysfunction. The four components were therefore interdependent. This model explains why the patient's collapse was substantially more serious than simple dehydration.

The diagnostic gap: investigating the source but missing the physiological consequences

The diagnostic challenge was not simply a failure to investigate the urinary symptoms. The patient was under active urological follow-up and underwent cystoscopy during the period of persistent macroscopic haematuria. The more important safety gap was that, despite approximately six weeks of visible haematuria, no full blood count or other blood-test assessment was performed during the ambulatory period. As a result, the systemic consequence of continued blood loss, namely progressive and ultimately profound anaemia, remained unrecognised until the patient presented after recurrent collapse, when his haemoglobin was found to be 50 g/L. This distinction is important: investigation of the anatomical source of haematuria does not necessarily identify the physiological consequences of continued bleeding.

Persistent macroscopic haematuria therefore created two clinical questions that needed to be considered in parallel: what was causing the bleeding, and what was the bleeding doing to the patient? The first question was being investigated through urological assessment and cystoscopy, whereas the second was insufficiently assessed. In an older patient with prolonged visible haematuria, recurrent collapse, and significant cardiovascular comorbidities, failure to identify severe anaemia may represent an important missed opportunity for earlier recognition of physiological deterioration. The clinical focus remained predominantly on the urinary symptoms, while the systemic consequences of the continuing haematuria were not recognised.

Diagnostic anchoring and premature closure

Diagnostic anchoring refers to the tendency to remain attached to an initial diagnostic impression despite subsequent information that should prompt reconsideration. Premature closure occurs when the diagnostic process effectively stops after an initial explanation has been accepted. Diagnostic errors in internal medicine are often multifactorial, and cognitive factors such as premature closure and faulty synthesis have been identified as important contributors. A systematic review of cognitive biases has also demonstrated a potential relationship between cognitive bias and diagnostic inaccuracy [16,17].

This case illustrates how anchoring can occur without a lack of clinical knowledge. UTI is a common and plausible explanation for urinary symptoms. Once that diagnosis was established, subsequent clinical care remained focused on the urinary problem, while the physiological consequences of six weeks of visible haematuria were not objectively assessed. The absence of an FBC during this period is particularly important. Without a haemoglobin measurement, the possibility of progressive anaemia could not be recognised. The eventual haemoglobin concentration of 50 g/L therefore represented not only a severe abnormality but also evidence of a late discovery of a potentially evolving physiological problem.

Management

The immediate management priority was correcting the life-threatening anaemia. Emergency packed red blood cell transfusion was therefore performed. Current international transfusion guidelines generally recommend a restrictive transfusion strategy for stable hospitalised adults, with consideration of transfusion at haemoglobin concentrations below 70 g/L and a threshold of around 80 g/L being reasonable in selected patients with pre-existing cardiovascular disease. This patient's haemoglobin concentration of 50 g/L was substantially below either threshold.

Transfusion decisions in patients with cardiovascular disease require particular care because both severe anaemia and excessive transfusion may have adverse consequences. The REALITY trial compared restrictive and liberal transfusion strategies in patients with acute myocardial infarction and anaemia and found that the restrictive strategy was non-inferior for the primary composite outcome, although a clinically important harm from the restrictive approach could not be excluded. The subsequent MINT trial further examined transfusion strategies in patients with myocardial infarction and anaemia. Its findings again showed uncertainty regarding the optimal transfusion threshold in acute myocardial infarction and supported individualised clinical decision-making.

In this patient, however, the haemoglobin concentration of 50 g/L represented extreme anaemia rather than borderline transfusion eligibility. Together with recurrent collapse and underlying cardiovascular vulnerabilities, this provided a strong physiological indication for urgent restoration of oxygen-carrying capacity. Fluid management also required careful consideration. The ambulance team had identified postural hypotension and initiated intravenous fluids. Volume optimisation was appropriate, but excessive fluid administration could have been hazardous in a patient with previous pulmonary oedema. The aim was therefore to restore adequate perfusion while repeatedly reassessing volume status and cardiac function.

The ECG abnormalities required urgent cardiology involvement. Because the patient had recurrent collapse in association with significant conduction disease and previous myocardial infarction, urgent specialist assessment for permanent pacing was arranged. The renal abnormalities prompted urgent nephrology involvement and transfer. The KDIGO guideline provides a structured approach to the diagnosis, staging, prevention, and treatment of AKI, including assessment of reversible causes and consideration of renal replacement therapy when clinically indicated. The patient's management therefore required a multidisciplinary approach involving emergency medicine, cardiology, nephrology, and urology. Treating only one component would have been insufficient because the physiological collapse resulted from the interaction of several abnormalities.

## Discussion

The central learning point from this case is that collapse should be interpreted physiologically rather than through a single diagnostic label. The presence of postural hypotension initially encouraged the diagnosis of dehydration. However, postural hypotension in older adults is frequently multifactorial and may result from volume depletion, medications, autonomic dysfunction, cardiovascular disease, or haemorrhage. The finding should therefore trigger investigation of its causes rather than automatically ending the diagnostic process.

In this patient, profound anaemia provided a major explanation for the reduced physiological reserve. A haemoglobin concentration of 50 g/L dramatically reduced oxygen-carrying capacity. Experimental evidence shows that when oxygen delivery falls sufficiently, tissue oxygen consumption can become supply-dependent and lactic acidosis may develop. The elevated lactate of 4.9 mmol/L was therefore compatible with significant physiological stress. However, lactate is not specific for infection or sepsis and should not be interpreted in isolation. In this case, the lactate had to be considered alongside profound anaemia, hypotension, severe renal dysfunction, and cardiac disease rather than as an isolated marker of infection [18].

This is relevant because the patient's original outpatient diagnosis was UTI. The presence of a recent antibiotic prescription could have made an infectious explanation more readily available for subsequent deterioration. However, the severe anaemia and cardiovascular abnormalities provided alternative explanations that required urgent attention. The cardiac component was equally important. The patient's ECG showed RBBB, left axis deviation, and a prolonged PR interval. Although the term "trifascicular block" is commonly used, the more clinically useful interpretation is that the ECG indicated significant conduction-system disease [19].

In a patient with recurrent collapse, previous STEMI, and conduction abnormalities, intermittent advanced AV block must be considered. NICE identifies conduction abnormalities as red flags following transient loss of consciousness and recommends urgent cardiovascular assessment in appropriate patients [20]. The renal component further indicated that the process was systemic. AKI is associated with adverse outcomes and can arise from haemodynamic disturbances such as hypoperfusion. In this patient, severe anaemia and postural hypotension provided plausible mechanisms for impaired renal perfusion. The severe metabolic acidosis provided additional evidence of physiological compromise. A pH of 7.25 and bicarbonate of 16.3 mmol/L reflected a significant acid-base disturbance. The raised lactate suggested systemic metabolic stress, although the precise contribution of hypoperfusion, reduced oxygen delivery, and other metabolic factors could not be determined from lactate alone.

The "quadruple-hit" concept therefore provides a useful framework. Hit 1 (haematological): profound anaemia reduced oxygen-carrying capacity. Hit 2 (haemodynamic): postural hypotension reduced effective circulating volume. Hit 3 (cardiac or electrical): previous STEMI and conduction disease limited the ability to compensate. Hit 4 (renal or metabolic): severe AKI and acidosis reflected end-organ injury and further reduced physiological reserve. The key problem was not simply the presence of four abnormalities. It was the interaction between them.

Severe anaemia required increased cardiac output. Conduction disease potentially restricted the heart's ability to increase rate and maintain adequate output. Postural hypotension then further reduced venous return and perfusion. The kidneys, dependent on adequate renal blood flow, became vulnerable to hypoperfusion. Once AKI and metabolic acidosis developed, the patient's physiological reserve became even smaller [21]. This created a self-reinforcing cycle of deterioration. Several practical lessons emerge from this case.

First, persistent macroscopic haematuria should remain an active clinical problem until appropriately explained. Although this patient was under active urological assessment, including cystoscopy, a diagnosis of UTI should not close the broader diagnostic pathway when visible haematuria persists. Second, the consequences of continuing bleeding should be considered alongside the cause of bleeding. In a patient with prolonged visible haematuria, clinicians should consider whether significant anaemia or renal dysfunction has developed. Third, the absence of an FBC during this patient's six-week ambulatory follow-up represented a major missed opportunity. The later haemoglobin concentration of 50 g/L shows that severe anaemia remained clinically unrecognised until presentation with physiological deterioration.

Fourth, postural hypotension should not automatically be equated with dehydration. In older adults, postural hypotension is often multifactorial and can reflect haemorrhage, medication effects, autonomic dysfunction, or cardiovascular disease. Fifth, an abnormal ECG should be integrated with the clinical context. RBBB, left axis deviation, and a prolonged PR interval in a patient with recurrent collapse and previous STEMI should not be dismissed as incidental. Sixth, severe AKI should be interpreted as a potential marker of systemic physiological compromise rather than simply an isolated renal abnormality.

Finally, clinicians should deliberately reassess an established diagnosis when new findings do not fit it. Diagnostic anchoring and premature closure are recognised contributors to diagnostic error. The safest approach is to ask periodically whether the original diagnosis still explains the patient's current presentation.

## Conclusions

This case report demonstrates how a seemingly common outpatient diagnosis can obscure a rapidly evolving multi-system emergency. A 95-year-old man with previous STEMI, pulmonary oedema, and hypertension was under ambulatory urological follow-up for six weeks of persistent macroscopic haematuria and had been treated for presumed UTI. No FBC or other blood tests were performed during these six weeks. The patient's haemoglobin was therefore not monitored despite continuing haematuria. He subsequently experienced three episodes of sudden collapse that were initially attributed to dehydration after a profound postural drop in blood pressure was identified. Hospital assessment revealed a haemoglobin concentration of 50 g/L, significant conduction-system abnormalities, severe AKI (creatinine 363 micromol/L, urea 39.4 mmol/L, and eGFR 12 mL/min), and metabolic acidosis (pH 7.25, lactate 4.9 mmol/L, and bicarbonate 16.3 mmol/L). The patient's deterioration can be understood as a "quadruple-hit" cardiorenal collapse in which severe anaemia impaired oxygen delivery, postural hypotension reduced effective perfusion, previous myocardial injury and conduction disease limited cardiovascular compensation, and renal hypoperfusion contributed to severe AKI and metabolic disturbance.

The most important patient-safety lesson is the delayed recognition of severe anaemia during prolonged ambulatory follow-up. The lack of FBC or other blood tests meant that the physiological consequences of persistent haematuria were not identified until the patient presented with collapse. This illustrates that diagnostic delay can occur not only through anchoring on an incorrect diagnosis but also through failure to reassess an evolving problem and failure to perform appropriate investigations when the clinical trajectory warrants them. The case reinforces three important clinical principles. First, postural hypotension should not automatically be attributed to dehydration. Second, persistent visible haematuria in an older adult requires appropriate investigation even when UTI has been diagnosed or treated. Third, recurrent syncope with significant conduction abnormalities and structural heart disease requires urgent cardiovascular assessment. Most importantly, clinicians should be prepared to reopen the diagnostic process when the patient's trajectory no longer fits the original explanation. A familiar diagnosis such as UTI may explain one symptom while failing to explain the patient's overall physiological deterioration. In patients with persistent haematuria, proactive safety-netting and appropriate blood-test assessment may identify severe anaemia and renal dysfunction before physiological compensation fails.
